# Integrated space–frequency–time domain feature extraction for MEG-based Alzheimer’s disease classification

**DOI:** 10.1186/s40708-021-00145-1

**Published:** 2021-11-02

**Authors:** Su Yang, Jose Miguel Sanchez Bornot, Ricardo Bruña Fernandez, Farzin Deravi, KongFatt Wong-Lin, Girijesh Prasad

**Affiliations:** 1 Department of Computer Science, Swansea University, Swansea, UK; 2 Intelligent Systems Research Centre, School of Computing, Eng & Intel. Sys, Ulster University, Derry-Londonderry, Northern Ireland UK; 3 School of Engineering and Digital Arts at the University of Kent, Canterbury, UK; 4 Centre for Biomedical Technology, Technical University of Madrid, Madrid, Spain

**Keywords:** Multi-domain, Magnetoencephalography, Biomarkers, Spatio-temporal features, Alzheimer’s disease, Mild cognitive impairment

## Abstract

Magnetoencephalography (MEG) has been combined with machine learning techniques, to recognize the Alzheimer’s disease (AD), one of the most common forms of dementia. However, most of the previous studies are limited to binary classification and do not fully utilize the two available MEG modalities (extracted using magnetometer and gradiometer sensors). AD consists of several stages of progression, this study addresses this limitation by using both magnetometer and gradiometer data to discriminate between participants with AD, AD-related mild cognitive impairment (MCI), and healthy control (HC) participants in the form of a three-class classification problem. A series of wavelet-based biomarkers are developed and evaluated, which concurrently leverage the spatial, frequency and time domain characteristics of the signal. A bimodal recognition system based on an improved score-level fusion approach is proposed to reinforce interpretation of the brain activity captured by magnetometers and gradiometers. In this preliminary study, it was found that the markers derived from gradiometer tend to outperform the magnetometer-based markers. Interestingly, out of the total 10 regions of interest, left-frontal lobe demonstrates about 8% higher mean recognition rate than the second-best performing region (left temporal lobe) for AD/MCI/HC classification. Among the four types of markers proposed in this work, the spatial marker developed using wavelet coefficients provided the best recognition performance for the three-way classification. Overall, the proposed approach provides promising results for the potential of AD/MCI/HC three-way classification utilizing the bimodal MEG data.

## Introduction

There has been a rapid development of machine learning (ML) techniques applied to electroencephalography (EEG) and magnetoencephalography (MEG) data for the diagnosis of dementia, and particularly its most common form, the Alzheimer’s disease (AD) [1]. MEG data often can be derived from two complementary sensors, magnetometers and gradiometers [2, 3]. Clinically, the stages of AD progression can be classified under the categories of being healthy, AD’s prodromal stage of mild cognitive impairment (MCI), and healthy control (HC) [1]. However, as noted by other researchers, classification of the three AD, MCI and HC classes using MEG is quite challenging, while the usage of other modalities, such as EEG and fMRI for this particular problem is more commonly seen in the field [4, 5].

In one of the studies by Suk et al. [4], a series of binary classifications for AD/HC and MCI/HC was conducted, using a deep learning technique based on a stacked autoencoder. A publicly available database was employed for their work, which contained multiple modalities such as magnetic resonance imaging (MRI) and positron emission tomography (PET) scans. The $A{\upbeta }_{42}$, *t*-tau and *p*-tau were extracted as features from 51 AD patients, 99 MCI patients and 52 HCs. With respect to the MCI individuals, 43 of which progressed to AD, while 56 of the remaining did not progress to MCI in 18 months. Their proposed method was able to reach detection rates (against HC) of 95.9%, 85.0%, and 75.8%, for AD, MCI, and MCI-converter classes. Using the same database, Li et al. [6] proposed a model based on the restricted Boltzmann machine, for the abnormal detections (i.e. HC against AD, MCI and MCI-converters) using both MRI and PET modalities. In total, four classes were explored in their study. Based on the leave-one-out cross-validation (LOOCV), a recognition rate of 91.4% for AD vs. HC was reported. MCI vs. HC and MCI vs. AD produced much degraded classification performances, yielding 77.4% and 70.1%, respectively. In addition, given that MCI is a prodromal phase of AD and its symptoms are mixed, the classification performance between the two types of MCI participants (MCI vs. MCI-converters) was only 58.1%. It should be noted that despite the good performances achieved, this work is also based on two-class classifications.

Using only MRI data, Fang et al. [7] explored the binary classifications of AD vs. HC and MCI vs. HC, tested on a relatively large (greater than 100) population. The employed database included 190 HC, 305 MCI and 133 AD participants. A Gaussian discriminant analysis-based (GDA) algorithm was implemented for the classification. Measures of 25 labelled cortical regions were used to generate two separate feature vectors (one per hemisphere), and a tenfold cross-validation was used to evaluate the algorithm for parameter tuning, using 80% of the data. In this scenario, a high recognition rate of 96.54% was reported for the classification between AD and HC, while the performance degraded to 90.26% for MCI vs. HC. Further, the optimized GDA model was fed for a hold-out cross-validation test using the remaining 20% of data; an accuracy of 93.10% for AD vs. HC classification was reported; the classification of MCI and HC achieved recognition rate of 80.61%.

A recent study on extracting region-specific (hippocampus and entorhinal cortex) biomarkers for AD dementia and amnestic MCI detections, appears to indicate that the variability of MCI types could further increase the challenge for classification [7]. Based on the quantitative MRI for feature extraction, the sensitivities for amnestic MCI (aMCI) vs. HC was 79.0%, and AD vs. HC reached 94.5%; their respective specificities were 85.6% and 86.1%. In total, 25 subjects with aMCI and 14 patients with AD dementia were classified against 62 HC subjects. A series of image-based features, including T1-weighted imaging, T2-weighted imaging, T2 relaxometry and diffusion tensor imaging were fed into a support vector machine (SVM) with 2nd order polynomial kernel for classification.

It is noticed that EEG studies that attempt to diagnose AD rarely use multi-class classification or combine the EEG and MEG modalities. For instance, Fiscon et al. [8] conducted a series of binary classification using a 19-electrode EEG recording device (standard 10–20 sensor placement). Data from 100 subjects were obtained, of which 14 subjects were HCs, 49 subjects were patients with AD, and the remaining 37 were MCIs. A continuous segment of 180 s in the centre of the recording for each subject was employed for feature extraction. The conventional Fourier coefficients were fed to three classifiers for comparison, namely a SVM with 2nd order polynomial kernel, decision trees and rule-based classifiers. The best recognition rates were provided by the decision tree classifier: AD vs. HC 73%, MCI vs. HC 90% and AD vs. MCI 80%. Later, the same research team improved the classification accuracy performance using a series of wavelet-based features (mean, standard deviation and power spectral density of the wavelet coefficients), reporting classification rates of 83% for AD vs. HC, 92% for MCI vs. HC and 79% for AD vs. MCI [5].

A few general points can be drawn from the aforementioned literature. First, most studies indicate that the classification between AD and HC had better performance, in comparison to the MCI vs. HC problem. This is to be expected, given the longitudinal nature of MCI, such a transitional stage from healthy to advanced dementia may last for a few years. Moreover, although MCI is mostly considered a prodromal stage of AD, it may be also related to other dementia disorders or comorbidities [9], which increased the challenge for accurate detection. Thirdly, as one major limitation, the three-way classification (i.e. AD vs. MCI vs. HC) has been rarely explored and reported, reflected these studies. Clinically, it is important to be able to discriminate multiple classes, so the proper clinical intervention (specific for a particular type of brain disorder) can be implemented in a timely manner. Indeed, multi-view feature learning for MCI and dementia diagnosis has been explored recently [10–12]. However, to the best of our knowledge, three-class classification analysis fully utilizing the two complementary MEG modalities (magnetometers and gradiometers) has yet to be thoroughly explored and reported in the literature. Given the excellent spatio-temporal resolution of MEG signals, its exploitation in MEG-based classification of MCI/AD against HC is quite promising [12, 13].

Therefore, the major focus of this work is to address the three-class classification problem using a series of newly developed wavelet-based biomarkers (features) to leverage the unique advantages of MEG-related signals. To alleviate the impact of the differences between participants, a novel bimodal (magnetometer and gradiometer) recognition system is proposed, in which the classification outcomes are fused at the score-level to achieve an improved and more robust recognition performance.

The rest of the paper is organized as follows: Section 2 is devoted to introducing the proposed approach, which is further divided into two subsections to illustrate the system pipeline and highlight the characteristics of the proposed new algorithm, respectively. In Sect. 3 preliminary investigations are reported aimed at optimizing the system parameters and exploring the sensitivity to the region of interest (ROI). A series of comparative results on the three-class classification are presented in Sect. 4 to emphasize the effectiveness of the proposed features/markers. To leverage the information captured by the magnetometer and gradiometer modalities, a new method for score-level fusion is further proposed for an improved recognition performance. The proposed approach is designed to improve the recognition rate by preserving biomarkers from only the effective region of interest. To compare the performance of the proposed method with the existing literature, the conventional two-way classification is also explored in this section. In Sect. 5, we discuss the advantages and limitations of two popular approaches for system evaluation, namely leave-one-out cross-validation and Monte Carlo random sampling cross-validation (MCRSCV) [14]. An improved evaluation method, with arguably more objective characteristics, is then proposed and tested in this study. The comparisons between the proposed evaluation approach and the other two conventional approaches are explored. The conclusions and suggestions for future work are presented in Sect. 6.

## Methodologies and the proposed features

A wavelet-based feature extraction method is proposed in this section to harness multi-domanial (time/frequency/space) information of the MEG data. Unlike conventional signal processing approaches which try to address the problem from time or/and frequency perspectives, the proposed new approach is trying to also leverage the spatial information of the signals that is available, thanks to the high-resolution data provided by the employed MEG systems. Coupled with the time–frequency analysis of wavelet decomposition, the novel method proposed here is performing a spatial–time–frequency analysis on the MEG data for a three-way classification on AD, MCI and HC participants.

This section is divided into two parts. Section 2.1 describes the overall system design; the expert system pipeline starts from raw brain signals and terminates with the final classification decision. Section 2.2 is devoted to highlighting the new approach based on using a two-dimensional wavelet decomposition, and will focus on the proposed new segmentation approach for MEG signal patching, inspired by image segmentation techniques.

### System design

**Fig. 1:**
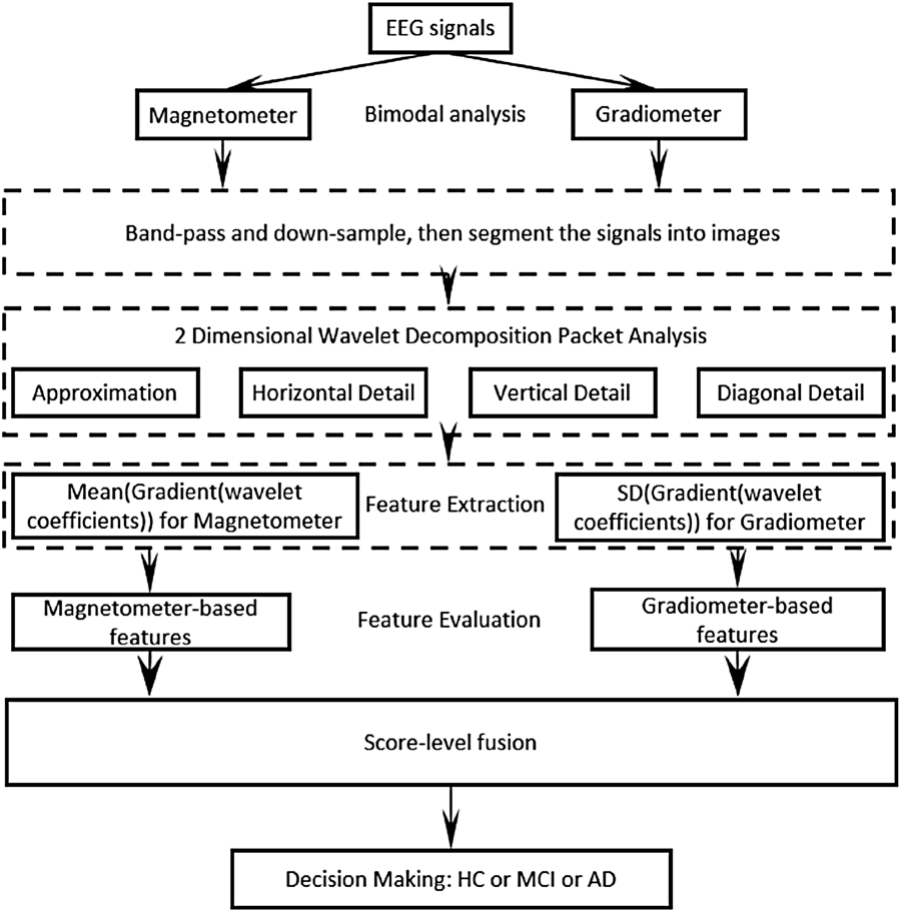
Flow diagram of the proposed bimodal three-class recognition system

All the brainwave signals in this study were captured using the 306-channel Elekta MEG systems [2, 3], equipped with two major types of sensor: 102 magnetometers and 204 planar gradiometers (102 derivative along latitude and 102 derivative along longitude directions [15]). Magnetometers consist of a single coil which measures the magnetic flux perpendicular to its surface. Planar gradiometers consist of a "figure-of-eight"-type coil configuration. The measured signal is the difference between the two loops of the "eight" [15]. The data collected by magnetometer and gradiometer sensors may be considered as two different modalities and were processed separately in this work (Fig. 1), due to the different meaning and measuring scale.

The original sampling frequency of the signals captured through Elekta system was 1000 Hz. To extract the frequency band of interest and avoid the possibility of aliasing, the signal was band-limited to 200 Hz for fast computation using a 4th order low-pass Butterworth filter, which was further band-passed to 80 Hz for better capturing the signal trend. Conventionally, for EEG signals, only up to 60 Hz is made use for brain activation analysis, whereas MEG signal may be capable of revealing useful content contained within higher frequency ranges [16].

The low-passed signals were firstly segmented into 10-s (2000 samples) epochs. A number of sensors from a selected region of interest (ROI) were grouped to produce a series of *M* × *N* matrices that can be interpreted as 2D images. For each of these images, *M* denotes the number of samples per epoch and *N* represents the number of sensors for each ROI. The images were fed into a series of two-dimensional wavelet packet decomposition (WPD) filter banks, where they were used for the preliminary feature extraction [17]. The details of this approach are presented in Sect. 2.2.

After the preliminary feature extraction, the resulting wavelet coefficients were processed separately for magnetometer and gradiometer channels. For the magnetometer, the statistical mean of the WPD coefficients for each image was computed, whereas for the data from gradiometers, the standard deviation (SD) of the image after the derivative of the wavelet coefficient was computed (Fig. 1). Computing the mean and SD for the final stage of feature extraction is based on a few well-established approaches reported in [5, 18], and serves for both dimension reduction and effective feature extraction. The resulting features were then fed to a 3-nearest neighbour (3-NN) and a quadratic Bayes normal classifier (QBNC) to generate confidence scores. These two classifiers are the best performed models after a range of tests, include SVM and neural nets with optimized parameters. The selection of these classifiers is based on preliminary investigations on a number of mainstream classifiers, including back-propagation neural networks, support vector machine with polynomial kernel, and linear discriminate analysis. Due to the limited data available, deep learning networks were not employed in this study. To leverage the two modalities (magnetometer- and gradiometer-based), a score-level fusion was used to achieve a further improvement of the recognition performance.

### Two-dimensional WPD for MEG image

One novelty of the proposed approach is concatenating multiple MEG signals into an image for a two-dimensional wavelet multivariate analysis. Rather than conducting feature extraction for each sensor individually, multiple nearby sensors (follow the standard MEG sensor labelling system [19]) are concatenated to form an MEG image, with its horizontal direction representing the spatial information (sensor, *N*), its vertical direction indicating the time information (epoch, *M*).

Compared to EEG, MEG systems are usually equipped with larger number of sensors (roughly 300 vs. 100), and such dense sensor distribution facilitates a higher spatial resolution for the measured brainwave. However, signals captured by sensors are conventionally treated independently for feature extraction, disregarding the fact that nearby sensors are picking similar activities from underlying neuronal sources, and combining multiple nearby sensors may enhance the measurement of the underlying activity. Ignoring the spatial relationships of sensors, therefore, makes it difficult to benefit from the advantage of the high spatial resolution provided by MEG sensor matrix.

**Fig. 2:**
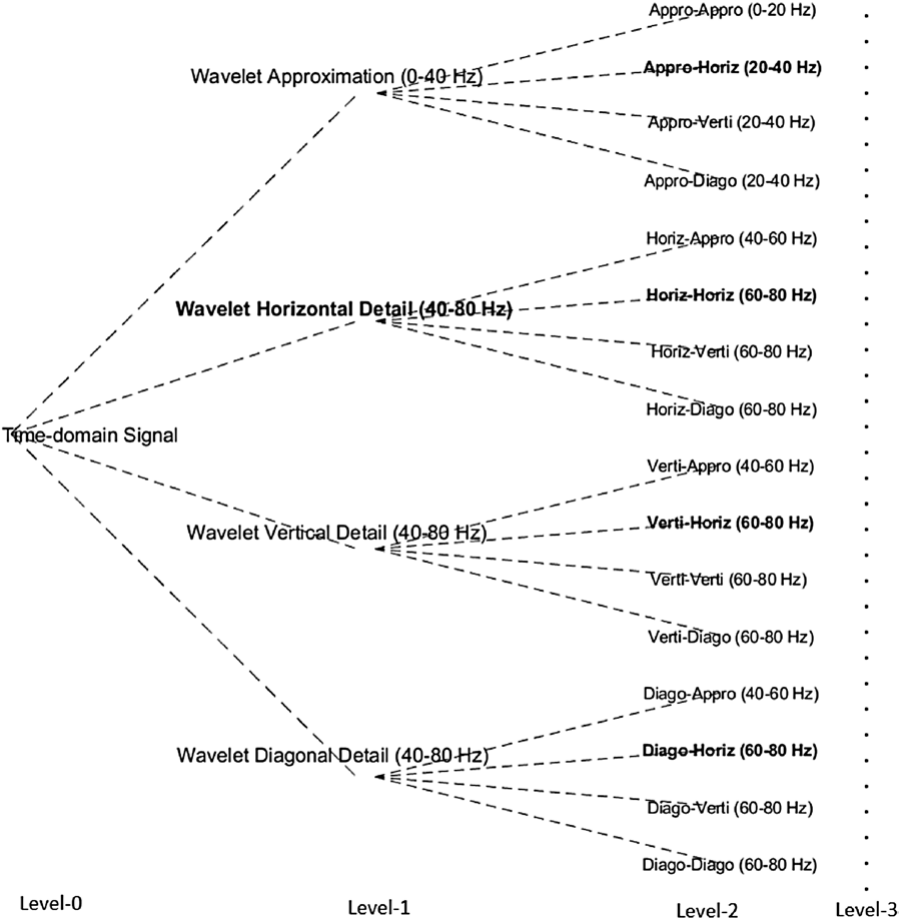
The two-dimensional wavelet packet decomposition tree; the best-performing coefficients are highlighted with bold. The decomposition is performed up to Level-3, which has been simplified for neat illustration

In this study, the two-dimensional wavelet packet decomposition (2D-WPD) is employed for analysing the characteristics of signals, obtained through magnetometers and gradiometers. For each MEG image, the 2D-WPD initially produces four nodes of sub-band coefficients for each level of decomposition in the wavelet domain, namely: approximation, horizontal detail, vertical detail and diagonal detail coefficients [17, 20]. The approximations reflect the low-frequency part (shape) of the signal; the remaining three “detail” nodes extract the high-frequency part of the signal along three directions (domains): horizontal detail reflects the time; vertical detail reflects the space and diagonal detail reflects both the time and spatial information. Multi-resolution analysis can also be achieved across overlapped frequency ranges as the decomposition level is increased (see Fig. 2). For an image defined by ${ }f\left( {x,y} \right)$, the 2D-WPD can be expressed as:

1 $$ f\left( {x,y} \right) = \frac{1}{{\sqrt {MN} }}\mathop \sum \limits_{m} \mathop \sum \limits_{n} W_{\varphi } \left( {j_{0} ,m,n} \right)\varphi_{{j_{0} ,m,n}} \left( {x,y} \right) + \frac{1}{{\sqrt {MN} }}\mathop \sum \limits_{i = H,V,D} \mathop \sum \limits_{{j = j_{0} }}^{\infty } \mathop \sum \limits_{m} \mathop \sum \limits_{n} W_{\psi }^{i} \left( {j,m,n} \right)\psi_{j,m,n}^{i} \left( {x,y} \right), $$

where (in Eq. 1) $W_{\varphi }$ and $W_{\psi }^{i}$ are defined below:

2 $$ W_{\varphi } \left( {j_{0} ,m,n} \right) = \frac{1}{{\sqrt {MN} }}\mathop \sum \limits_{x = 0}^{M - 1} \mathop \sum \limits_{y = 0}^{N - 1} f\left( {x,y} \right)\varphi_{{j_{0} ,m,n}} \left( {x,y} \right), $$

3 $$ W_{\psi }^{i} \left( {j,m,n} \right) = \frac{1}{{\sqrt {MN} }}\mathop \sum \limits_{x = 0}^{M - 1} \mathop \sum \limits_{y = 0}^{N - 1} f\left( {x,y} \right)\psi_{j,m,n}^{i} \left( {x,y} \right), i = \left\{ {H,V,D} \right\}, $$

in which the symbol $j_{0}$ indicates an arbitrary starting scale, $W_{\varphi } \left( {j_{0} ,m,n} \right){ }$ defines an approximation (Eq. 2) of $f\left( {x,y} \right)$ at scale ${ }j_{0}$, $W_{\psi }^{i} \left( {j,m,n} \right)$ add horizontal, vertical and diagonal details (Eq. 3) for scales ${ }j > j_{0}$. These four functions are expressed by Eqs. 8–9, which comprise the scaled (by *j*) and shifted (by *m*, *n*) by the wavelet functions (Eqs. 4–7) which can be used to synthesize the original signal [21]:

4 $$ \varphi \left( {x,y} \right) = \varphi \left( x \right)\varphi \left( y \right), $$

5 $$ \psi^{H} \left( {x,y} \right) = \psi \left( x \right)\varphi \left( y \right), $$

6 $$ \psi^{V} \left( {x,y} \right) = \psi \left( x \right)\varphi \left( y \right), $$

7 $$ \psi^{D} \left( {x,y} \right) = \psi \left( x \right)\varphi \left( y \right), $$

8 $$ \varphi_{j,m,n} \left( {x,y} \right) = 2^{\frac{j}{2}} \varphi \left( {2^{j} x - m,2^{j} y - n} \right), $$

9 $$ \psi_{j,m,n}^{i} \left( {x,y} \right) = 2^{\frac{j}{2}} \varphi \left( {2^{j} x - m,2^{j} y - n} \right), i = \left\{ {H,V,D} \right\}. $$

When the two-dimensional analysis is performed, wavelet approximation captures both the low-frequency content and the time domain trend of multiple signals simultaneously. In this implementation, the detail coefficients reflect each MEG image patch from three aspects: horizontal detail for spatial information, vertical detail for temporal information, and diagonal for both the spatial and time domain contents. As shown in Fig. 2, the three detail measurements retain the high-frequency part of the signal within each decomposition level. Combining time, frequency and spatial domains, the features based on this extraction method may, therefore, be more effective in solving challenging classification problems than conventional approaches. In the next section, this feature extraction method will be applied to address the three-class classification problem for distinguishing AD, MCI and HC subjects.

## Experimental analysis

In this study, we combined the data from two MEG databases for analysis. The AD data were collected in the Intelligent Systems Research Centre at Ulster University (Londonderry, UK) [3]. Participants were recruited from a specialist memory clinic following a full assessment including history, physical examination, neuropsychological testing and neuroimaging. Diagnoses were made according to a standard clinical criterion. Healthy controls were instructed to complete an assessment to ensure there was no evidence of cognitive impairment. The MCI/HC data were collected in the Hospital Universitario de San Carlos (Madrid, Spain) [2]. In total, the MEG data were collected from elderly participants with comparable age range: 7 AD, 20 MCI and 20 HC. All MEG data were collected in the resting-state condition, 5-min sessions for AD and 3-min sessions for MCI/HC participants. All participants were right-handed as verified with the Edinburgh Handedness Inventory (Oldfield, 1971) further details of which can be found in [22].

Data from both sources were processed offline using the temporal extension of the signal space separation (tSSS) algorithm [23] through MaxFilter 2.2 with a window length of 10 s, and a correlation threshold of 0.9. The same algorithm was used, in conjunction with the instantaneous head position obtained from the Head Position Indicator coils, to correct the movement of the participant during the recording. In order to guarantee same amount of data are used as for the HC and MCI classes, for the data of subjects in the AD database, we purposely kept only the central 3 min of the recordings. This data was combined with the data from 14 randomly selected subjects from the MCI/HC database (seven participants per class), hence formed a three-class database with a balanced class frequency for training and testing. All the data from 21 participants of the combined AD/MCI/HC database went through the pre-processing steps introduced in Sect. 2.1.

### Preliminary investigations

A few preliminary investigations were conducted to explore and enhance the performance of the proposed recognition system. The effectiveness of feature type, sensor type and sensor location with respect to the classification performance are discussed in this section. The sensor location map is divided into ten conventional regions of interest (ROIs) [19] and the data from each ROI were processed separately.

As it was stated in the previous section, the 2D-WPD analysis resulted in four different nodes per decomposition level, where each node captured specific information from the original signal in the wavelet domain. The approximation coefficients reflect the overall trend of the signal. The horizontal node extracted spatial information of the signals from the stacked sensors; the vertical node potentially better reflected the signal fluctuations over time. Further, the diagonal node combined information from both spatial and temporal domains, consequently providing higher-order features.

### Feature analysis

The effectiveness of features derived from different wavelet nodes at each level was explored and the results are listed in Table 1. For the sake of clarity and simplicity, only the data from left-frontal region were used for this analysis, which has been found most effective. The listed recognition accuracies are the averaged results obtained by an improved cross-validation approach, which will be detailed in Sect. 5. Features were extracted from magnetometers and gradiometers separately. Note that depending on the type of modality, the final step for feature extraction is different in order to obtain the best performance (see Fig. 1 for the different approach per modality).

**Table 1:** Feature analysis with respect to the two modalities; these sampled results are for the LF region

Modality	Magnetometer		Gradiometer	
Final feature	**Type-Mean** (%)	Type-SD (%)	Type-Mean (%)	**Type-SD** (%)
Approximation	64.30	48.89	51.96	72.02
Horizontal detail	**66.52**	44.70	52.30	**85.93**
Vertical detail	63.26	38.22	55.85	74.00
diagonal detail	65.81	38.67	34.67	77.70

The optimal features and performances are highlighted in bold

**Fig. 3:**
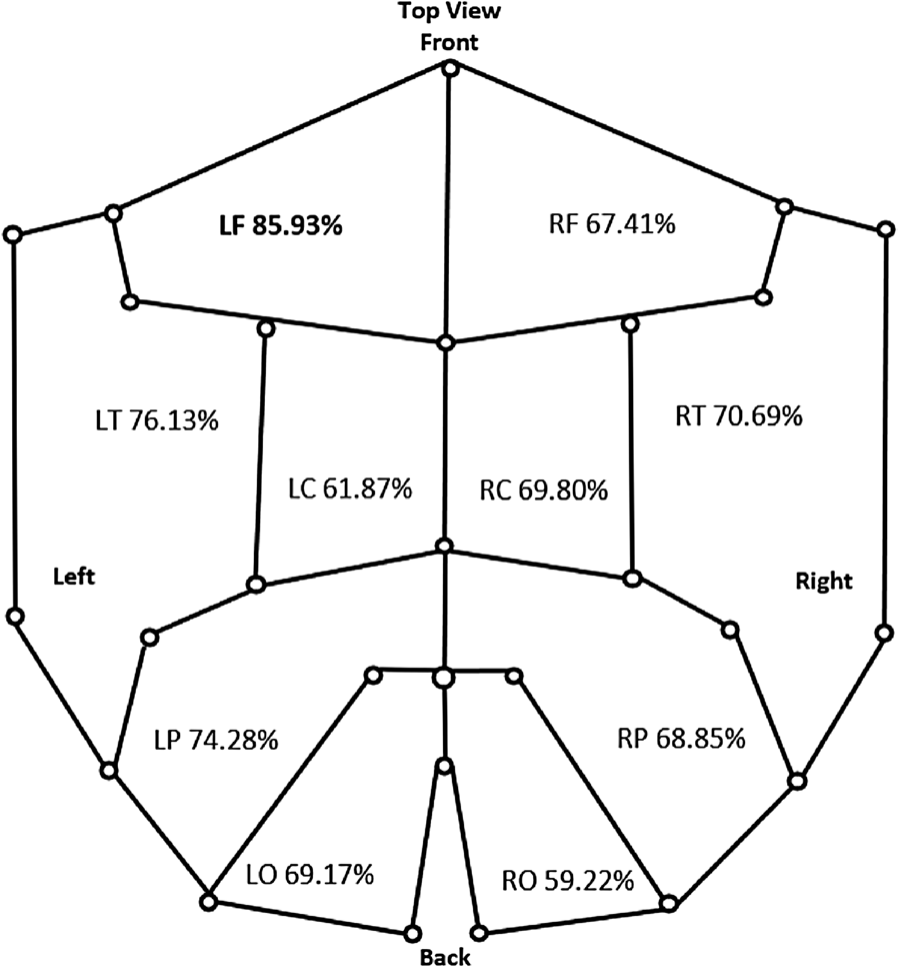
ROI separation and the respective effectiveness for the three-class classification, note the six sensors in the centerline are excluded for this symmetric analysis. Each label indicates the mean accuracy for the three-class classification, using the hybrid LOO–MCRS cross-validation approach for evaluation. Note these results are obtained through the magnetometer–gradiometer score-level fusion

Table 1 provides classification results for the LF region, which is best performed ROI according to our preliminary study (see Fig. 3). Type-Mean indicates computing the mean of the coefficient gradients for the final feature extraction and Type-SD indicates computing the standard deviation of coefficient gradients. It is found from Table 1 that the Type-Mean is more effective for magnetometer signals, whereas Type-SD is found to be more effective for gradiometer signals. With respect to the four types of wavelet nodes after WPD, features derived from horizontal detail coefficients have been found the most effective for both modalities, which appears to be indicating that the signal obtained by magnetometers and gradiometers indeed contain rich spatial information. Features based on the remaining three type of decomposition nodes revealed less accurate classification performance, compared to features derived from the horizontal detail wavelet components. It is worth note that, directly concatenating the features from different modalities has been found cannot boost the performance. Based on the results in Table 1, features computed using the horizontal detail coefficients were, therefore, preserved in the follow-up analysis for further system optimization.

### ROI analysis

In order to locate the most effective region of interest for the three-class classification, the individual ROIs were explored separately. As shown in Fig. 3, the scalp may be divided into 10 typical ROIs with left and right hemispheres [19], namely left/right frontal (LF/RF), left/right temporal (LT/RT), left/right central (LC/RC), left/right parietal (LP/RP) and left/right occipital (LO/RO) ROIs. This subdivision was adopted for our study and the mean cross-validation results are itemized for each ROI to indicate their respective effectiveness in discriminating the three classes.

Features derived from LF lobe (22 sensors) have been found to provide the highest mean recognition rate (85.93%), which is more than 9% higher than the second highest accuracy, obtained from the LT lobe. This is probably due to the potential disorder of this region which might be associated with memory impairment [24], which is one typical effect of dementia such as AD. Interestingly, it is found that in general the ROIs in the left hemisphere tend to provide much better averaged classification performance (6% ~ 10% higher in averaged accuracy), compared to those located in the right hemisphere; the only exception is the central region ROI pair, which demonstrates a reversed trend on the effectiveness for recognition performance (Fig. 3).

## Score-level fusion and comparative analysis

Based on the preliminary investigations conducted in Sect. 3, a few important observations can be made: (1) the features derived from gradiometers modality have been found to be more effective than those from the magnetometers; (2) the resulting wavelet horizontal detail coefficients after two-dimensional WPD, produced the features with the highest recognition rate; (3) the frontal left region is the most effective lobe for the AD–MCI–HC classification problem in this implementation. In this section, we continue exploring a solution for leveraging the advantages of combining the two modalities for further improvement in classification performance.

The features derived from wavelet coefficients are results of multi-resolution analysis; the frequency ranges up to 80 Hz were analysed in three levels of scale, each with a different resolution. Only the horizontal detail coefficient nodes (spatial information) were kept for further feature extraction, as highlighted in Fig. 2, given that they provided the highest performance (see Table 1).

**Fig. 4:**
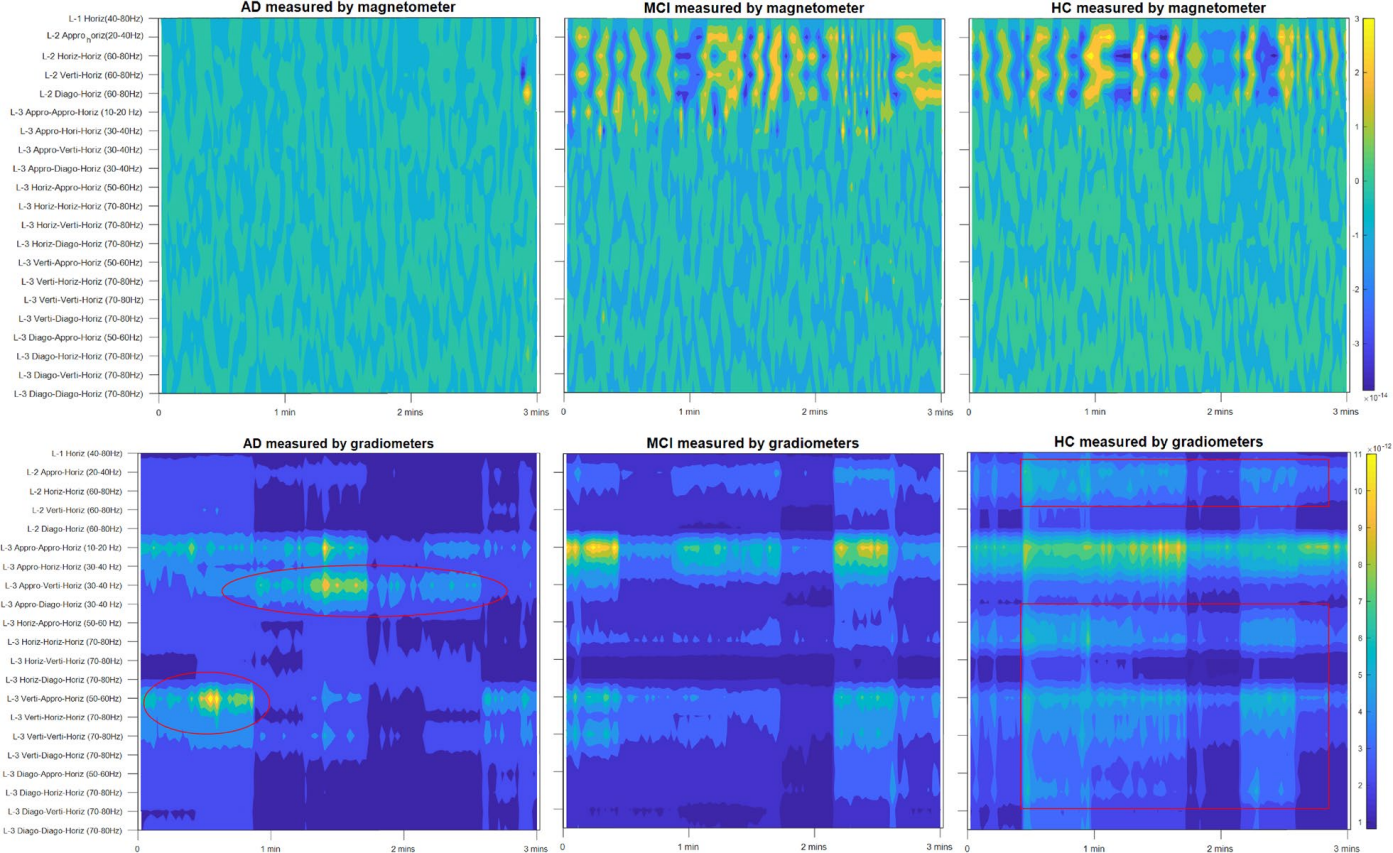
Feature heat maps for two modalities, magnetometer (first row) and gradiometer (second row), using data from all the participants of three classes: AD, MCI and HC. L-1, L-2 and L-3 indicate the level of decomposition. The horizontal axis indicates the recording length

Figure 4 presents heat maps of the extracted markers. In this figure, the features for both the magnetometer (first row) and gradiometer (last row) are shown for the three different classes (AD, MCI and HC). In total, 21 nodes of horizontal detail coefficients were selected for feature extraction, which formed a feature matrix with 21 dimensions which form the vertical indices of the heat maps. While further inspecting Fig. 4, it is clear that for the magnetometer modality, AD is clearly distinguishable from the other two classes, in particular within the level-2 resolution scale. However, is hard to distinguish between MCI and HC in all resolutions and frequency ranges using the magnetometer-based features. This may explain why the best classification rate obtained using the feature computed from magnetometers was only around 66% (shown in Table 1).

The heat maps of three classes from gradiometer-based features, on the other hand, appear to be much more separable. The HC features are found in general with higher values across three resolutions and multiple frequency ranges, particularly in the high-frequency bands (highlighted in red squares), which have traditionally been ignored for analysis in EEG signals. Both the MCI and AD features show smaller values across multiple bands and resolution scales, which may indicate their relatively less active status of the brain compared to the HC. However, the features for L-3 and gamma band “L-3 Appro-Verti-Horiz (30–40 Hz)” and “L-3 Verti-Appro-Horiz (50–60 Hz)” showed much higher values for AD than MCI class, as seen in the encircled parts of Fig. 4. These observations may moderately justify the better recognition performance obtained using the gradiometer modality (about 84%), in comparison to the magnetometer-based features.

In order to comprehensively explore the effectiveness of the proposed method toward each different group detection, the confusion matrix generated using the best features from the left-frontal region is illustrated in Table 2. From the results it is clear that, though the three-way classification is a more challenging problem, the proposed features and method shown excellent performance in separating the AD from MCI and HC groups. For the employed database, the healthy control group is more inclined to be misdiagnosed as with MCI, compared to the other way around misdiagnosis.

**Table 2:** Confusion matrix for the three-way classification performance using the features derived from the left-frontal region

Test observation: *n* = 54, each class with 18	Predicted AD	Predicted MCI	Predicted HC
Actual AD	18	0	0
Actual MCI	0	16.5	1.5
Actual HC	0	6.1	11.9

The results are obtained using the optimal feature engineering approach, i.e. the horizontal detail coefficients with Type-SD, from the gradiometer modality

An additional investigation was conducted to compare the proposed method with other published reports on AD and MCI detections. The recognition results for three binary detection scenarios are shown in Table 3. Evidently, even using only the magnetometer modality, the proposed method outperforms most of the reported results, which may indicate the potential advantages of MEG-related features against EEG and MRI in spatio-temporal and frequency domains. However, the MCI vs. HC classification of the proposed feature appears to be relatively less effective than the other two classification cases.

**Table 3:** Comparison on the modality and recognition scenarios, the magnetometer and gradiometer are referring to the MEG-related modalities based on the proposed method

Modality	AD vs. MCI (%)	AD vs. HC (%)	MCI vs. HC (%)
Magnetometer	94.72	95.56	52.78
Gradiometer	98.89	98.33	81.67
MRI [6]	70.1	91.4	77.4
MRI [32]	91.7	95.8	93.8
EEG [33]	79	83	92

Results after modality fusion are shown in Table 4

By closely inspecting Fig. 4, it was noticed that for the magnetometer modality, the discrimination of AD vs. MCI (or HC) appears to be very high. While using the gradiometer modality, the discrimination of HC vs. (AD) (or MCI) is found to be quite high. Naturally, it is logical to consider leveraging the advantages of both modalities, hence further improvements on recognition performance may be achieved. In this work, we propose to perform a novel score-level fusion of the two modalities to achieve this purpose.

Due to its close relationship with the fusing of the scores produced by modality-sensitive classifiers, it is important to remember the data organization for the proposed approach: all the participants’ data were concatenated and further divided into 378 epochs (10 s per epoch). This optimal epoch size is identified based on our preliminary exploration of this parameter, as well as our previous studies on EEG signals [25]. Therefore, for each participant 18 epochs of data were available and the output for the classifier per subject, was an 18 × 3 score matrix, i.e. 18 observations (one for each epoch) with three classes.

After normalization, each observation is a vector containing three scores (sum up to 1), which indicates the confidence of the observation for class assignment in question.

Because of a low performance improvement observed in a pilot study (about 1%), the resulting score matrix was not directly used for bimodal fusion. In this work, we firstly computed the mean probability of all the observations for each class. The product fusion rule was then performed, i.e. the mean probability score from each class (Eqs. 10 and 11) was obtained by:

10 $$ p\left( {\overline{{U_{j} }} } \right) = \frac{{\mathop \sum \nolimits_{i = 1}^{i = n} U_{i,J} }}{n}, $$

11 $$ p\left( {\overline{{V_{j} }} } \right) = \frac{{\mathop \sum \nolimits_{i = 1}^{i = n} V_{i,J} }}{n}. $$

The most effective magnetometer-based (Type-Mean) and gradiometer-based features (Type-SD) are used for the fusion (Table 1), denoted by $$\overline{{U_{j} }}$$ and $$\overline{{V_{j} }}$$, respectively,$$ i \in \left[ {1,126} \right]$$ indicate the 126 observations for each class and $$ J \in \left[ {1,3} \right]$$ represents the three classes in question. Note the actual number of observations used for training may be varying depending on the evaluation approach adopted, features of the remaining observations will be used for evaluation (to be further described in the following section). Equations 10, 11 compute the average scores produced by the modality-driven classifiers 3-NN and QBNC, respectively. The final scores using the product rule for bimodal fusion was computed using Eq. 12, the fused score was then produced using Eq. 12 as $$ W_{J}$$:

12 $$ W_{J} = p\left( {\overline{{U_{j} }} } \right)*p\left( {\overline{{V_{j} }} } \right),J \in \left[ {1,3} \right]. $$

**Fig. 5:**
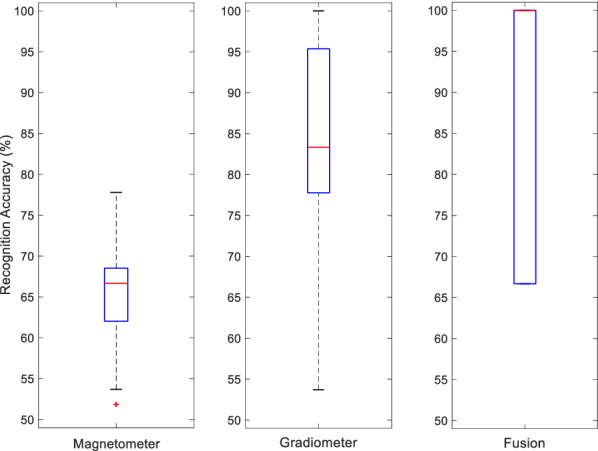
Comparison between two modalities and the effectiveness of their score-level fusions: Megn mean accuracy (3-NN) 65.43%, SD 0.0514; Grad mean accuracy (QBNC) 85.93%, SD 0.1089; Fusion mean accuracy: 88.33%, SD 0.1598

Figure 5 shows the comparison of magnetometer-based, gradiometer-based and score-fused bimodal systems for the three-way classification. It was found that the mean recognition accuracy (using the proposed evaluation described in Sect. 5) of the bimodal score-fusion system received more than 4% of improvement (88.33%). The variance of the bimodal system performance is also found to have increased. This may be due to the considerable difference in recognition rate between the two modalities.

Due to the limited data availability for training and testing, a modified evaluation scheme has been adopted for this study to make the most use of the available data.

Seven iterations are performed in order to separate the participants, using the conventional LOOCV scheme [26, 27]. Another popular testing approach namely Monte Carlo random sub-sampling cross-validation (MCRSCV) can resolve the iteration limitation of LOOCV. With this approach, the data are randomly split into training and validation sets, and for each such split, test is performed using the validation set. As a sampling with replacement approach, this process can be repeated as many times as needed to reveal the variance of the performance and, therefore, achieve potentially more representative results. One of the shortcomings of this approach, however, is that the random splitting mechanism may lead to non-selection of some instances for validation, whereas other instances may be selected for testing multiple times.

Another even more severe issue of using MCRSCV for evaluation, particularly for the problem of interest in this work, is the data from the same participants who have been exposed to the classifier, may also be considered for testing, i.e. though the features for testing is different, but they are from the same recording or participant. As a result, the MCRS cross-validation performance would be biased and tend to be overly optimistic.

## Evaluation and discussion

In this work, we proposed a hybrid method to combine the advantages of LOOCV and MCRSCV. To avoid the possible bias of the performance from MCRSCV, and increase the number of iterations for cross-validation, the feature sets are reconfigured using the following steps:

1. Each feature set per class is split into a few subsets, within each subset contains all the features from one individual. Here, it is seven subsets, each subset accounts for the data from one participant.
2. One subset is randomly selected as the preliminary evaluation set; the remaining six subsets are used to form the training set.
3. Randomly picking up 50% of the features from the preliminary evaluation set to form the final evaluation set, which is then tested by the classifier trained using the previously generated training set in Step (2).
4. Step (1)–Step (3) are repeated multiple times to guarantee the data from all the participants per class get test chance (repeated 100 times in this study, in order to generate the representative boxplot). The averaged result is then computed.

With this hybrid LOO–MCRS evaluation approach, the possible performance bias of MCRSCV for the implementation in this work is avoided, as there is no possibility of using the data from the same participant for testing. It also resolved the limitation of repetitions for LOOCV while the number of folds is small, as in each repetition only 50% of the data from one participant were randomly picked up for test. To demonstrate the sensitivity of the results to the evaluation scheme used, a comparison of the three evaluation approaches is presented in Table 3.

Table 4 shows the results using the three evaluation approaches discussed in this section, the recognition rates were obtained using the best performed features derived from the wavelet horizontal detail coefficients. It is found that while using MCRSCV approach for system evaluation, the results tend to be better than those obtained through LOOCV, which is in line with the previous discussion: MCRSCV tends to provide overly optimistic evaluation results by tolerating the measurements from the same participant(s).

**Table 4:** Comparison of the evaluation methodologies; the proposed hybrid approach is referred as LOO–MCRS

Modality	LOOCV (%)	MCRSCV (%)	LOO–MCRS (%)
Magnetometer	65.25	67.52	65.19
Gradiometer	85.93	91.57	83.87
Score fusion	89.84	93.76	88.33

The results are the mean values of 100 times repetitions

Repeating the tests 100 times, the proposed hybrid approach provided comparable or slightly worse recognition rates than the conventional LOOCV. It should be noted that, certain outliers could slightly bias the mean performance while using LOOCV for performance measurement. In this study, using the data from one particular participant, one excessively high recognition rate of 90% was achieved from using gradiometer modality, which lifted the averaged accuracy obtained using LOOCV. The LOO–MCRS approach, with a lot more random and unbiased tests, may indicate an arguably more objective evaluation of the system than LOOCV.

The p-values of the recognition rates for each evaluation scheme were computed, in order to explore the statistical significance of the performances in this work (Table 3). As a rule of thumb, A *p*-value less than 0.05 is usually considered statistically significant. The final *p*-values for HC vs. MCI, HC vs. AD and MCI vs. AD were 0.048, $3.1 \times 10^{ - 5}$ and $1.2 \times 10^{ - 8}$, respectively. This analysis was conducted by performing the Kruskal–Wallis H test [28] with the Holm–Bonferroni correction [29] for this multiple testing problem.

## Conclusion and future work

In this work, we presented a bimodal recognition algorithm to explore the effectiveness of three-class (AD vs. MCI vs. HC) classification based on MEG modalities. The MEG-derived gradiometer modality in particular, appears to have the potential for superior performance while using the proposed two-dimensional wavelet-based features for the classification tasks. This newly proposed feature leverages the spatial, temporal and frequency characteristics of the signals obtained by magnetometers (102 sensors) and gradiometers (204 sensors); and holds the promise of improved recognition performance through a modality fusion at the score-level.

One of the major challenges in this preliminary research is the access to large amount of clinical data, as well as the segmentation of the AD stages in more details. Indeed, if without focusing on single region for MCI/AD detection, combining the multiple effective regions may potentially boost the performance [30]. However, attributable to certain physiological/physical similarities in terms of the brainwave among participants [31], using the principle of transfer learning to grasp the similar patterns from different participants for the brain malfunction detection, could be a promising research direction. Another interesting direction is to combine EEG and MEG for analysis. The data collection for these two types of signals can be conducted simultaneously. The two modalities are based on different data capturing principles, improved recognition performance could be expected, since the two modalities reflect the characteristics of brainwave from different perspectives. Further exploration could be conducted on the recognition of the types of MCIs, such as Amnestic vs. Non-Amnestic MCI, Single Domain vs. Multiple Domain MCI.

## Data Availability

Data not available due to ethical/legal restrictions.
